# Supplementary material for: Disability and pain after lumbar surgery–group-based trajectory analysis
Source: PLoS One. 2025 Jan 9;20(1):e0313528. doi: 10.1371/journal.pone.0313528 (PMC11717237; doi:10.1371/journal.pone.0313528)
Supplement: S1 Fig — (DOCX) [file pone.0313528.s001.docx]

S3 Fig. Trajectories of changes in pain and disability after surgery comparing fusion and no-fusion techniques.

Repeated measures: #1 – baseline, #2 – 3 months, #3 – 1 year and #4 – 2 years
