# Supplementary material for: Disability and pain after lumbar surgery–group-based trajectory analysis
Source: PLoS One. 2025 Jan 9;20(1):e0313528. doi: 10.1371/journal.pone.0313528 (PMC11717237; doi:10.1371/journal.pone.0313528)
Supplement: S1 Table — (DOCX) [file pone.0313528.s002.docx]

S1 Table. Goodness of fit of group-based trajectory analysis models. The chosen models are shown underlined

| Number of clusters | Regression orders | Smallest group | | BIC^1^ | AIC^2^ | Smallest APP^3^ |
| --- | --- | --- | --- | --- | --- | --- |
|  |  | N | % |  |  |  |
| Disability (ODI) | | | | | | |
| 1 | 3 | 1451 | 100 | 10211 | 10197 | 1 |
| 2 | 3 / 3 | 252 | 17 | 9986 | 9957 | 0.86 |
| 3 | Variance matrix is nonsymmetric or highly singular | | | | | |
| Back pain (VAS) | | | | | | |
| 1 | 3 | 1451 | 100 | 10871 | 10856 | 1 |
| 2 | 3 / 3 | 457 | 32 | 10754 | 10725 | 0.81 |
| 3 | 3 / 3 / 3 | Insignificant p-value even for linear model | | | | |
| 3 | 2 / 3 / 3 | 179 | 12 | 10706 | 10651 | 0.70 |
| 4 | Variance matrix is nonsymmetric or highly singular | | | | | |
| Leg pain (VAS) | | | | | | |
| 1 | 3 | 1451 | 100 | 10640 | 10626 | 1 |
| 2 | 3 / 3 | 535 | 37 | 10529 | 10510 | 0.77 |
| 3 | 3 / 3 / 3 | 104 | 7 | 10515 | 10471 | 0.70 |

^1^ BIC = Bayesian Information Criterion, ^2^ AIC = Akaike information criterion, ^3^ APP = Smallest average posterior probability; ^4^ – Statistical significance was not achieved even for linear models
