# Supplementary material for: Disability and pain after lumbar surgery–group-based trajectory analysis
Source: PLoS One. 2025 Jan 9;20(1):e0313528. doi: 10.1371/journal.pone.0313528 (PMC11717237; doi:10.1371/journal.pone.0313528)
Supplement: S2 Table — (DOCX) [file pone.0313528.s003.docx]

S2 Table. ODI scores and pain severity at different waves by trajectory groups

| Study waves | Group #1: | | | Group #2: | | |
| --- | --- | --- | --- | --- | --- | --- |
|  | Mean | 95% CI | | Mean | 95% CI | |
| Groups based on disability level | | | | | | |
| ODI score, points |  |  |  |  |  |  |
| Baseline | 36.68 | 35.48 | 37.89 | 57.59 | 55.22 | 59.96 |
| 3 months | 15.04 | 12.98 | 17.10 | 41.56 | 38.13 | 44.98 |
| 1 year | 13.66 | 11.78 | 15.54 | 43.55 | 40.46 | 46.63 |
| 2 years | 14.88 | 12.23 | 17.52 | 46.52 | 42.73 | 50.32 |
| Groups based on back pain severity | | | | | | |
| Pain severity, points |  |  |  |  |  |  |
| Baseline | 51.92 | 47.93 | 55.91 | 68.03 | 62.84 | 73.23 |
| 3 months | 16.05 | 12.84 | 19.27 | 44.72 | 39.28 | 50.16 |
| 1 year | 16.75 | 13.66 | 19.83 | 58.18 | 52.75 | 63.61 |
| 2 years | 20.55 | 15.81 | 25.29 | 65.98 | 59.37 | 72.59 |
| Groups based on leg pain severity | | | | | | |
| Pain severity, points |  |  |  |  |  |  |
| Baseline | 57.68 | 53.19 | 62.17 | 68.97 | 64.07 | 73.87 |
| 3 months | 15.33 | 11.97 | 18.68 | 48.80 | 42.11 | 55.49 |
| 1 year | 17.53 | 13.64 | 21.41 | 58.69 | 53.10 | 64.29 |
| 2 years | 21.54 | 15.85 | 27.22 | 64.29 | 57.45 | 71.14 |
