# Supplementary material for: Disability and pain after lumbar surgery–group-based trajectory analysis
Source: PLoS One. 2025 Jan 9;20(1):e0313528. doi: 10.1371/journal.pone.0313528 (PMC11717237; doi:10.1371/journal.pone.0313528)
Supplement: S3 Table — (DOCX) [file pone.0313528.s004.docx]

S4 Table. Descriptive characteristics of sample by fusion vs. no-fusion techinques

| Characteristic | No fusion | Fusion | Total | *p*-value^a^ |
| --- | --- | --- | --- | --- |
|  | n=898 (61.9%) | n=553 (38.1%) | n=1,451 (100.0%) |  |
| Age, years | 69 (11.3) | 63 (12.5) | 67 (12.1) | <0.001 |
| Sex |  |  |  |  |
| Men | 473 (53%) | 185 (33%) | 658 (45%) | <0.001 |
| Women | 425 (47%) | 368 (67%) | 793 (55%) |  |
| Preoperative pain duration |  |  |  |  |
| <3 months | 388 (43%) | 179 (32%) | 567 (39%) | <0.001 |
| >3 months | 510 (57%) | 374 (68%) | 884 (61%) |  |
| Leg pain, points | 64 (26.1) | 63 (26.7) | 64 (26.3) | 0.924 |
| Back pain, points | 56 (27.8) | 63 (24.7) | 59 (26.8) | <0.001 |
| Oswestry Disability Index, % | 41 (16.4) | 44 (17.4) | 42 (16.9) | 0.003 |
| Body mass index, kg/m^2^ | 29 (4.9) | 29 (4.9) | 29 (4.9) | 0.915 |

^a^ Chi2 test or ANOVA
