# Supplementary material for: Disability and pain after lumbar surgery–group-based trajectory analysis
Source: PLoS One. 2025 Jan 9;20(1):e0313528. doi: 10.1371/journal.pone.0313528 (PMC11717237; doi:10.1371/journal.pone.0313528)
Supplement: S4 Table — (DOCX) [file pone.0313528.s005.docx]

S5 Table. Relative risk ratios (RRRs) of being classified to a short-term improvement group (reference is long-term improvement group) comparing fusion vs. no fusion techniques. RRRs are adjusted for age and sex.

| Comparators | RRR | 95% CI | |
| --- | --- | --- | --- |
| Oswestry Disability Index |  |  |  |
| No fusion |  |  |  |
| Women vs. men | 1.01 | 0.71 | 1.43 |
| Age (older vs younger) | 0.94 | 0.66 | 1.33 |
| Pain duration >=3 months vs. <3 months | 1.09 | 0.77 | 1.55 |
| BMI ≥30 vs. BMI <30 | 1.47 | 1.04 | 2.08 |
| Fusion |  |  |  |
| Women vs. men | 0.77 | 0.50 | 1.20 |
| Age (older vs younger) | 1.45 | 0.95 | 2.21 |
| Pain duration >=3 months vs. <3 months | 1.11 | 0.71 | 1.75 |
| BMI ≥30 vs. BMI <30 | 1.41 | 0.92 | 2.14 |
| Back pain |  |  |  |
| No fusion |  |  |  |
| Women vs. men | 1.23 | 0.93 | 1.63 |
| Age (older vs younger) | 0.86 | 0.65 | 1.14 |
| Pain duration >=3 months vs. <3 months | 1.34 | 1.01 | 1.78 |
| BMI ≥30 vs. BMI <30 | 1.19 | 0.90 | 1.58 |
| Fusion |  |  |  |
| Women vs. men | 0.87 | 0.59 | 1.28 |
| Age (older vs younger) | 1.02 | 0.70 | 1.49 |
| Pain duration >=3 months vs. <3 months | 1.13 | 0.76 | 1.68 |
| BMI ≥30 vs. BMI <30 | 1.85 | 1.27 | 2.67 |
| Leg pain |  |  |  |
| No fusion |  |  |  |
| Women vs. men | 1.35 | 1.02 | 1.78 |
| Age (older vs younger) | 1.08 | 0.82 | 1.43 |
| Pain duration >=3 months vs. <3 months | 1.00 | 0.75 | 1.32 |
| BMI ≥30 vs. BMI <30 | 1.03 | 0.78 | 1.36 |
| Fusion |  |  |  |
| Women vs. men | 0.88 | 0.61 | 1.26 |
| Age (older vs younger) | 1.23 | 0.87 | 1.75 |
| Pain duration >=3 months vs. <3 months | 1.03 | 0.71 | 1.47 |
| BMI ≥30 vs. BMI <30 | 1.58 | 1.12 | 2.22 |
