# Supplementary material for: Disability and pain after lumbar surgery–group-based trajectory analysis
Source: PLoS One. 2025 Jan 9;20(1):e0313528. doi: 10.1371/journal.pone.0313528 (PMC11717237; doi:10.1371/journal.pone.0313528)
Supplement: Attachment — Submitted filename: Response to Reviewers.docx [file pone.0313528.s006.docx]

Professor Shabnam ShahAli, Ph.D.

Academic Editor

PLOS ONE

Dear Professor ShahAli

Thank you for the opportunity to revise our manuscript PONE-D-24-24037 entitled "Disability and pain after lumbar surgery – group-based trajectory analysis”. We greatly appreciate the time and effort you and the reviewers have dedicated to improving our work. Please find submitted the revised manuscript and a detailed, point-by-point response to the reviewers' comments.

On behalf of all the authors

Konsta Koivunen

Responses to the comments made by the Editor

Comment 1

Please ensure that your manuscript meets PLOS ONE's style requirements,

including those for file naming.

Response 1

We have now ensured that the manuscript meets the style requirements.

Comment 2

In the online submission form you indicate that your data is not available for proprietary reasons and have provided a contact point for accessing this data. Please note that your current contact point is a co-author on this manuscript. According to our Data Policy, the contact point must not be an author on the manuscript and must be an institutional contact, ideally not an individual. Please revise your data statement to a non-author institutional point of contact, such as a data access or ethics committee, and send this to us via return email. Please also include contact information for the third party organization, and please include the full citation of where the data can be found.

Response 2

Dear Editor

The data used in this study were part of electronic patient record kept by the wellbeing services county of Southwest Finland. The data were delivered to the research team by the IT-services as an anonymous dataset. The raw data cannot be passed on to other parties. Limited dataset used in this particular analysis can be delivered by a corresponding author on a reasonable request. Additional requests regarding the data availability can be addressed to the ethical board of the wellbeing services county of Southwest Finland [*eettinen.toimikunta@varha.fi*](mailto:eettinen.toimikunta@varha.fi). Could you please inform us if this explanation is sufficient. If not, then we are ready to discuss any other acceptable solution.

Responses to the comments made by the Reviewer #1:

Comment 1

What is your logic for analyzing and categorizing patients according to their leg pain, back pain, etc.? Is it based on the literature or did you select them exploratively? Please clarify this in the objectives of your study and also in the method.

Introduction, p6: Please explain the objectives of the study in more detail with the rationales.

Response 1

We have now modified the objectives paragraph of introduction as follows:

*“The objective of this study was to identify groups exhibiting distinct trajectories within the broader cohort of patients undergoing lumbar spine surgery and to determine whether certain factors may be associated with the probability of being classified into a particular group. The common goal of lower back surgery is to alleviate radiating lower extremity pain in particular. This study also aimed to find out whether the change in the intensity of low back pain followed the change in lower extremity pain or whether the trajectories of these changes were different postoperatively. The registry data used in this study included both surgeries where fusion techniques were used and surgeries without fusion. It has been assumed here that the indications for surgery in these two groups might differ substantially. Therefore, changes in pain intensity were also analyzed for these two surgical subtypes as a sensitivity test.”*

Comment 2

Most of the study's supporting data are necessary to understand the study's results. It's better to provide them in the main text or as a figure or table.

Response 2

The main text contains now six figures and tables. We will be happy to move our supplemental material into the main text. However, we are not certain if this move will not exceed the common practice in PlosOne. The Editor may decide if this move can be done.

Comment 3

Abstract, p2, line 30: What does RRR stand for?

Response 3

We have now rewritten the sentence as follows:

*“Sex and age were not associated with being classified into short-term improvement groups, but obesity was associated for all three-factor variables with relative risk ratios (RRR) varying from 1.26 (95% CI 1.02 to 1.56) to 1.45 (95% CI 1.10 to 1.90).”*

Comment 4

Material and methods, p7, line 115: Change 'logit regression model' to 'logistic regression model'.

Response 4

We have now made the change as suggested.

Comment 5

Please rewrite the statistical analysis section of the manuscript. It is not clearly defined and raises so many questions for a reader.

Statistical analysis, p8, line 127: Why did you set the cut-off value to 10%?

Response5

We have now rewritten the “statistical analysis” section as follows:

*“The logical chain of GBTA analysis employed here was as follows. The first important question was how many groups (clusters) should be included in the analysis. The number of different trajectories can vary from one (the change in the average of the entire data set) to the n number of the sample (the trajectory is analyzed for each individual person). Another important choice was the shape of the regression curve (linear, quadratic, cubic, etc.). Based on these two issues, a model that would meet certain following requirements was preferred. There are no commonly accepted exact recommended selection criteria for the number of trajectories. It greatly depends on a sample size. Here the limit was set that none of the clusters should be smaller than 10% of the sample size. This cut-off was arbitrary and it was based on the sample size and the experience of the authors. As additional confirmation, another criterion was set - in no cluster should the average posterior probability (APP) be lower than 0.7. Regarding the degree of the regression curve, a common practice is to try models of different degrees and use in the analysis the highest possible degree, which produces a statistically significant result (p<0.05). The goodness of fit of the selected model was assured based on additional statistics: the Bayesian Information Criterion (BIC) and the Akaike Information Criterion (AIC) were calculated for the different combinations of the numbers of clusters and the regression degrees making sure that their values were closer to zero compared to models based on a smaller number of clusters or other regression degrees. Trajectory analysis was repeated separately for the ODI and the severity of leg and back pain. The models chosen and their goodness of fit are presented in S1 Table.*

*A two-cluster model demonstrated the best fit for changes in disability severity and leg pain. While a three-cluster model demonstrated an acceptable fit for back pain, the smallest average posterior probability for this model was close to a pre-agreed cut-off of 0.7 and the size of the smallest group was close to a pre-agreed cut-off of 10%. In order to ensure the easiness of interpretation of the results, further analysis was conducted using two-cluster models for all three-factor variables – disability, back pain, and leg pain.*

*The sensitivity test was performed by comparing those who underwent fusion (NAG61, NAG62, NAG63, NAG66, NAG67) vs. no-fusion (ABC36, ABC56, ABC66) surgery.*

*After identifying clusters, the probability of group membership was calculated based on sex, age, the leg and back pain duration before surgery, and obesity. These probabilities were expressed as RRR along with 95% confidence intervals (95% CI). All the analyses were conducted using Stata/IC Statistical Software: Release 18, College Station (StataCorp LP, TX, USA).”*

Comment 6

Discussion, p15, line 241: What does PROMs stand for?

Response 6

We have now rewritten the sentence as follows:

*“In 2021, Jamjoom et al. reported in a systematic review that the placebo effect could last up to six months after spinal surgery and it can be seen in patient-reported outcome measures (PROMs).”*

Comment 7

Discussion, p17, line 257: Please explain the probable reasons for these controversies?

Response 7

We have now rewritten this part of the discussion as follows:

*“The evidence of such a relationship has, however, been controversial. In their systematic review, Ghobrial et al. have stated that obesity is not related to higher post-operative VAS-measured back pain, leg pain, or functioning measured with ODI in populations undergoing non-instrumented lumbar spinal surgery according to minimal clinical important difference (MCID). However, in that review by Ghobrial et al, it was found that although overweight subjects had a significant improvement according to the MCID value, they still had greater pain both at baseline and post-operatively in almost all studies. Some other studies have also stated that, even though obesity is associated with more postoperative complications, even overweight people may benefit from surgery. However, Onyekwelu et al.reported that although overweight people benefited from surgery, normal weight people benefited more. Additionally, Lingutla et al. reported that problems arose in their study due to the non-standard definition of obesity and the limited availability of data. Also, the study by Divi et al. had a small sample size. Even more, Rihn et al. reported that obese patients may benefit more from surgery compared to nonoperative treatment than normal weight patients. On the other hand, this result could be explained by the fact that the response of overweight patients to nonoperative treatment was poor.”*

Comment 8

Discussion, p17, line 267-268: What do you mean by “The register did not contain data on spinal surgery prior to the follow-up”?

Response 8

We apologize for this typo. We have now rewritten this sentence as follows:

*“The data included information from patients who have undergone only one back surgery. However, this restriction only applied to data after the start of registry data collection. It is therefore possible that there were individual patients who underwent more than one operation, if the operations had been performed before the registry data started to be collected. This is a commonly known weakness of register data, a  truncation at the beginning of follow-up, which complicates the differentiation between prevalent and incident cases.”*

Comment 9

Please define groups in Figure1, 2 &3.

Response 9

We have now added the following footnote to all three figures:

*“Group #1 – long-term improvement group; group #2 – short-term improvement group”*

Responses to the comments made by the Reviewer #2:

Comment 1

Through the whole text please insert space between the statement and the reference. Also please insert the reference just after the author's name when it is applicable (for example line 54 after the name Willems et al.)

Response 1

We have now made these changes.

Comment 2

Please insert reference for the statement in line 101 and 102. Please do the same for lines 125 and 126.

Response 2

We have now inserted the references.

Comment 3

I would suggest explaining central sensitization as an explanation of pain maintenance even after the surgery (line 263)

Response 3

We have now added a sentence as follows with the appropriate citation:

*“Prolonged pain maintenance even after the surgery could be explained by central sensitization. Prolonged chronic pain may result in functional, chemical and even structural changes in central nervous system. If so, then nervous system can be persistently in a high activity state and a person can experience pain even in the absence of nociceptive input, which has successfully been treated with surgery.*

Responses to the comments made by the Reviewer #3:

Comment 1

Abstract: The background section lacks clarity and cohesion, requiring improvement. Additionally, the English writing throughout needs clarity and coherence.

Introduction: While the study's aim is clear, the background section could benefit from better cohesion leading toward the final conclusions.

Response 1

We have now modified the text of the “background” paragraph of the abstract as follows:

*“Previous studies in lumbar spine surgery have mainly studied functioning and pain by comparing average scores from Patient Reported Outcome Measures (PROMs) at different time points. Less is known about these changes in different subgroups. It is self-evident that, while most patients may demonstrate trajectories of these changes close to the average one, some groups may follow more or less different trends. Also, it is unclear which preoperative factors may affect the probability of being classified into groups with different development trajectories of surgical outcome. The objective of this study was to identify groups exhibiting distinct trajectories within the broader cohort of patients undergoing lumbar spine surgery and to determine whether certain factors may be associated with a probability of being classified into a particular group.”*

We have now also modified the “objectives” paragraph of the introduction (hoping that this also clarified the background of the need for a study) as follows:

*“The objective of this study was to identify groups exhibiting distinct trajectories within the broader cohort of patients undergoing lumbar spine surgery and to determine whether certain factors may be associated with the probability of being classified into a particular group. The common goal of lower back surgery is to alleviate radiating lower extremity pain in particular. This study also aimed to find out whether the change in the intensity of low back pain followed the change in lower extremity pain or whether the trajectories of these changes were different postoperatively. The registry data used in this study included both surgeries where fusion techniques were used and surgeries without fusion. It has been assumed here that the indications for surgery in these two groups might differ substantially. Therefore, changes in pain intensity were also analyzed for these two surgical subtypes as a sensitivity test.”*

Comment 2

Methodology: Clarification is needed regarding the administration of questionnaires to patients. The rationale behind the wide timeframe (2-4, 11-13, and 23-25 months post-surgery) for data collection should be elaborated upon. Although previous data were utilized, the specific rationale for this timeframe should be explicitly stated. Furthermore, details on how missing data were handled are crucial for assessing the study's robustness.

Response 2

We have now added the following explanation to the Methods section:

*“Due to the retrospective study design, the timeframe (2-4, 11-13-, and 23-25-months post-surgery) for data collection has been determined by the clinical practice existed in the clinic – patients have visited the clinic at these time points for post-operative controls.”*

We have now expand the text of the “strength and weaknesses” paragraph of discussion as follows:

*“The present retrospective study lacked the possibility to deal with missing data as these had not been available for analysis. Many patients did not come to the control visits at the clinic. It is uncertain if the data were missing due to random chance or due to specific reasons, e.g., people with the greatest improvement have not appeared at their post-operative control appointments or the other way round – people dissatisfied with the results of surgery were not willing participate into follow-up. It has previously been suggested that missing data can substantially affect the precision of estimated change in PROMs scores from clinical registry data.*

Comment 3

The study did not explore other patient-reported outcome measures (PROMs) that could provide a more comprehensive assessment of surgical success (e.g., quality of life measures, return to work). This omission and its implications should be discussed in the Discussion section.

Response 3

We have now included the sentences as follows to the Discussion:

*“The study did not explore other potentially relevant PROMs that could provide a more comprehensive assessment of surgical success (e.g., improved quality of life or return to work). This limitation could create uncertainty concerning the overall improvement after surgery. It has been previously reported that back pain and disability may correlate in some situations only loosely Thus, persistent pain does not necessarily mean unchanged quality of life, perceived general health or ability to work.”*

Comment 4

Discussion: While the manuscript briefly compares findings with previous studies, a more comprehensive discussion integrating conflicting results and methodological differences across studies would enhance the interpretation of the findings.

Response 4

We have now rewritten sections of the Discussion as follows:

*“In agreement with the present findings, obesity has previously been associated with worse surgical outcome. Park et al. have observed the association between BMI≥30 and inferior surgical results in a cohort of 32,000 patients undergoing lumbar spinal surgery. The evidence of such a relationship has, however, been controversial. In their systematic review, Ghobrial et al. have stated that obesity is not related to higher post-operative VAS-measured back pain, leg pain, or functioning measured with ODI in populations undergoing non-instrumented lumbar spinal surgery according to minimal clinical important difference (MCID). However, in that review by Ghobrial et al, it was found that although overweight subjects have showed a clinically significant improvement according to the MCID value, they still had greater pain both at baseline and post-operatively in almost all studies. Some other studies have also stated that, even though obesity is associated with more postoperative complications, even overweight people may benefit from surgery. However, Onyekwelu et al. reported that although overweight people benefited from surgery, normal weight people benefited more. Additionally, Lingutla et al. reported that problems arose in their study due to the non-standard definition of obesity and the limited availability of data. Also, the study by Divi et al. had a small sample size. Even more, Rihn et al. reported that obese patients may benefit more from surgery compared to nonoperative treatment than normal weight patients. On the other hand, this result could be explained by the fact that the response of overweight patients to nonoperative treatment was poor.”*

And:

*“In this study, neither gender nor age increased the probability of being categorized into groups with a worse outcome. Similar results have also been obtained from previous studies. Donnarumma et al. reported that women may benefit less from lumbar decompression surgery. However, their study was conducted with a small sample size. According to the study by Murphy et al. older age increases the risk of complications, longer hospital stay, and discharge to a place other than home. However, their study did not address disability or pain.”*

Comment 5

The manuscript lacks exploration into potential mechanisms underlying observed associations (e.g., why higher BMI correlates with poorer outcomes). This mechanistic insight should be addressed in the discussion.

Response 5

We have now added the following text to the discussion:

*In 2016, Jackson et al. published a systematic review where the effects of obesity on spine surgery were discussed. In that review, it was reported that overweight people have a higher incidence of back pain, radiating pain and a higher rate of disk degeneration. In the review, possible causes of increased disc degeneration were found, for example, to be greater mechanical compression on the spine, chronic inflammation related to obesity and reduced blood flow to the disc. Being overweight has also been linked to a higher risk of complications in previous studies.*
